# Tracking down worldwide *Puccinia psidii* dispersal

**DOI:** 10.1186/1753-6561-5-S7-P14

**Published:** 2011-09-13

**Authors:** Rodrigo Neves Graca, Amy RossS-Davis, Ned Klopfenstein, Mee Sook, Tobin Peever, Phil Cannon, Janice Uchida, Acelino Couto Alfenas

**Affiliations:** 1 Universidade Federal de Vicosa, Brazil; 2 USDA Forest Service, USA; 3 Kookmin University, South Korea; 4 Washington State University, USA; 5 University of Hawaii at Manoa, USA

## Background

*Puccinia psidii* causes rust disease on many host species in the Myrtaceae [1]. First reported in 1884 on guava in Southern Brazil [2], the rust has since been detected on several myrtaceous in South America, Central America, Caribbean, Mexico, USA: in Florida, California, and Hawaii. More recently, *P. psidii* was reported in Japan infecting *M. polymorpha*[3]. Of special note is that a rust was found infecting Myrtaceae species in Australia, where the fungus was reported as *Uredo rangelii*, based on the tonsure found on the urediniospores surface. However, DNA sequence data did not differentiate that rust from *P. psidii*[4], and the same tonsure patch, was also observed on rust urediniospores collected from several host species in Brazil [unpublished data].We have hypothesed that *P. psidii* was introduced into Hawaii through California by trade of rust infected myrtaceous plants, and that *P. psidii* populations from South America are distinct from the rust populations that became estabilished in California and Hawaii.

## Material and methods

Fast-evolving microsatellite markers were used to determine genetic relationships among rust populations from South America, California and Hawaii. Such genetic markers allow inferences about the potential sources of pathogen introduction.

## Results

The eight microsatellite loci analyzed revealed 14 multilocus genotypes (MGs) within the 221 *P. psidii* isolates. Isolates collected on seven different hosts in South America presented distinct MGs. In contrast, all rust isolates collected on nine myrtaceous hosts in the Hawaiian Islands were composed of only a single, unique MG. The same unique MG observed in Hawaii, was also detected on isolates from two different hosts in California,indicating a common origin of the rust genotype found in Hawaii and California.The isolates were grouped using a principal coordinates analysis (PCA). The first two axes of the PCA plot explained 89% of the total observed variation, with the first axis explaining 50% and the second 39%. According to the PCA, the *S. cumini* group is the South American group that is most closely related to the Hawaiian and Californian groups.

## Conclusions

The MG comprising all isolates from Hawaii and California is distinct from the MGs found in South America so far, suggesting that the Hawaiian and Californian isolates did not come directly from South America. Isolates from Florida, Central America, and the Caribbean must be analyzed to better understand potential relationships with pathogen dispersion to Hawaii.
